# Evaluation of Solidified Wastewater Treatment Sludge as a Potential SCM in Pervious Concrete Pavements

**DOI:** 10.3390/ma15144919

**Published:** 2022-07-14

**Authors:** Ognjen Govedarica, Marina Aškrabić, Milica Hadnađev-Kostić, Tatjana Vulić, Branislava Lekić, Vladana Rajaković-Ognjanović, Dimitrije Zakić

**Affiliations:** 1 Faculty of Civil Engineering, University of Belgrade, Bulevar Kralja Aleksandra 73, 11000 Beograd, Serbia; ogovedarica@grf.bg.ac.rs (O.G.); branaj@grf.bg.ac.rs (B.L.); vladana@grf.bg.ac.rs (V.R.-O.); dimmy@imk.grf.bg.ac.rs (D.Z.); 2 Department of Basic Engineering Disciplines, Faculty of Technology, University of Novi Sad, 21000 Novi Sad, Serbia; hadnadjev@tf.uns.ac.rs (M.H.-K.); tvulic@uns.ac.rs (T.V.)

**Keywords:** wastewater treatment sludge, solidification, pervious pavement concrete, supplementary cementitious materials, lightweight concrete

## Abstract

Waste and recycled materials have recently been used in the construction industry to comply with the principles of circular economy and sustainable development. The aim of this paper is to examine the potentials of solidified wastewater treatment sludge (SWWTS) as a supplementary cementitious material (SCM) in the production of lightweight pervious concrete pavers (LWPCP) suitable for pedestrian trails and rooftops (green) that comply with EU standards. Detailed characterization of SWWTS was performed, in order to understand its properties related to application as SCM, which led to the conclusion that it may be applied only as a filler, having 89.5% of Ca(OH)_2_. After thorough characterization, LWPCP samples were prepared and testing of physical and mechanical properties was conducted. The research showed that partial replacement of cement with SWWTS led to the decrease of all mechanical properties, ranging between 3.91 and 5.81 MPa for compressive strength and 0.97 to 1.23 MPa for flexural strength. However, all of the investigated mixtures showed a value higher than 3.5 MPa, which was defined as the lowest compressive strength in the range of pervious concrete properties. The addition of SWWTS led to a slight decrease in bulk density of the mixtures and an increase in water absorption. This could be explained by the reduction in hydration products that would fill in the micropores of the matrix, since SWWTS showed no pozzolanic reactivity. Pore sizes that prevail in the tested binder matrices are in accordance with the results measured on ordinary pervious concrete (the largest fraction of pores had a diameter between 0.02 and 0.2 μm). Low thermal conductivity nominates produced pavers as potential rooftop elements.

## 1. Introduction

Urban development is usually coupled with a rapid increase in impervious surface area and a correlated reduction in rainfall infiltration. Consequently, the increase in stormwater runoff results in urban flooding that impacts many cities worldwide, on an almost annual basis. Pervious/permeable/porous (3P) paving is an essential sustainable urban drainage technique that uses low impact development (LID) strategies to recover pre-urbanization hydrology and manage urban stormwater in a distributed manner at the source [1]. 3P pavements are specifically designed to promote stormwater infiltration through the paving and base courses where it is filtered through the layers. Pervious concrete pavements (PCPs) are able to reduce the runoff volumes/rates and simultaneously improve the stormwater quality [2]. PCPs work as a filter that traps surface runoff pollutants and treats the stormwater through sedimentation, filtration, sorption and biological processes [3]. Therefore PCPs have proven to be an alternative for rainwater harvesting and groundwater recharge [4,5].

Mechanical behavior of the pervious concrete system is as important as its hydraulic properties. The mechanical quality of the system not only relies on the compressive strength of the pervious concrete but also on the bearing capacity of the soil beneath. Pervious concrete mixtures have been found to develop compressive strengths in the range of 3.5 to 28 MPa [6]. These are lower compressive strengths than with conventional concrete and will only support light traffic loadings incl. sidewalks, patios, bicycle roads, driveways for residential and light commercial use [7,8]. Design and PCPs technical requirements for light loadings are rarely standardized; however, certain standards in this regard have recently been established in the EU [9]. In addition to conventional pedestrian sidewalks, PCPs have been applied for green roof patios [10,11]. In order to minimize static roof loads, lightweight aggregate (expanded clay) has recently been applied for PCP production [12].

Waste and recycled materials have lately been used in the construction industry to comply with the principles of circular economy and sustainable development. PCP production consumes many natural materials (like sand and gravel) and relatively high quantities of cement, which significantly contributes to greenhouse gas (GHG) emission. Meanwhile, industrial and mining waste is increasingly generated worldwide. Even if properly managed, this waste still requires large plots of land for final disposal. Incorporating wastes into PCP production could not only lower cement production, but also address the issue of waste, which would directly trigger double benefits for the environment [10,13]. Processed wastewater treatment sludge (WWTS) has been introduced as a supplementary cementitious material (SCM) in concrete mixtures for PCP production [14]. Although mechanical properties of the final product may decline to a certain extent, it could still meet the technical requirements that comply with EU standards.

This study is set out to explore the potential of solidified WWTS as a supplementary cementitious material in the production of lightweight pervious concrete pavers (LWPCP) suitable for pedestrian trails and (green) rooftops that comply with EU standards.

## 2. Supplementary Cementitious Materials in Concrete Mixtures

One of the most common steps during the implementation of sustainable development principles within the construction industry is to reduce utilization of natural component materials used for the production of composites, such as concrete and mortars. The United Nations Environmental Program Sustainable Buildings and Climate Initiative established a technical working group in 2015 with a goal to review practical alternative technologies that would lead to lowering the cost and environmental impact of cement production [15]. The report identified two key areas with the most promising potential that could initiate the reduction in CO_2_ emission over the next 20 to 30 years in the cement and concrete materials sectors:

1. “Extending the use of supplementary cementitious materials (SCMs) in cement to further reduce clinker content, chiefly by developing technology for the combined addition of calcined clay and limestone.
2. Reducing concrete’s clinker content by improving mix designs that allow for increased filler content, which can be added either via the cement or directly during concrete mixing” [15].

The study also showed that the most common clinker substitutes are reactive products from other industries, such as granulated blast furnace slag and fly ash. Still, the most widespread supplementary cementitious material was shown to be almost inert limestone filler.

### 2.1. Limestone Filler

Limestone filler used in cement and concrete is usually produced by grinding of limestone in quarrying operations and it consists mainly of calcium carbonate. It is considered to be an inert filler material that improves the hydration rate of cement compounds and increases the strength at early ages [16]. Nevertheless, limestone powder can influence the behavior of cement pastes through physical and chemical effects. The physical effects trigger the modification of particle size distribution, dilution and heterogeneous nucleation [17]; whereas, due to its chemical effects, partial reactivity of limestone powder is possible, initiating the reactions with mono-sulfate and calcium-aluminate hydrate forming calcium mono-carbo-aluminate. Higher fineness of limestone powder would enhance these reactions [18].

Thongsanitgarn et al. [19] investigated the effect of limestone powder addition on compressive strength and setting time of limestone-cement pastes. They concluded that the compressive strength decreased with the increasing amount of limestone. Furthermore, it was shown that the increasing fineness of limestone leads to the increase in compressive strength values.

### 2.2. Wastewater Treatment Sludge

One of the waste materials that can be considered as a potential SCM or filler is the wastewater treatment sludge (WWTS) or drinking water treatment sludge (DWTS). Taking into account the ongoing increasing number of water treatment facilities, the search for alternative approaches regarding the sludge disposal is of essential importance. Up until recently, landfill disposal remains the most common sludge management solution that resulted in a substantial increase in costs and decrease in landfill capacity. In the period from 2015 to 2019, between 9.6 and 13.3 thousand tons per annum of urban wastewater treatment sludge was produced in Serbia, and more than 95% was disposed of in landfill sites [20]. 

The chemical composition, grain size distribution and pozzolanic activity of the sludge in question will greatly depend on the initial treated water and the type of processes applied during its treatment [21,22]. The physical and chemical characteristics of sewage sludge can vary depending on the design of each treatment plant, type of wastewater, time of year, climate, etc. [23,24]. It is shown that incinerated sewage sludge ash usually has a similar chemical composition to other waste materials with pozzolanic properties (coal fly ash, for example), with high proportions of SiO_2_, Al_2_O_3_ and CaO [25,26]. 

A not so often used posttreatment method of WWTS is solidification [27], which is applied in only several WWTPs throughout Europe. This technology is a physical and chemical oxido-reduction process that involves the reaction of wastes with additives based on calcium-oxide and calcium-hydroxide. The product of these reactions is inert material and steam. According to [27,28], the technology is recognized by the EU as the best available technology for treating selected wastes from the oil and petrochemical industries, pharmaceutical wastes, food processing wastes and wastewater treatment sludge. The product obtained from this treatment technology is a white-gray inert granular powder that can be disposed of in nonhazardous waste landfill [27,29]. Nakic et al. [27] were investigating the possibility of using SWWTS obtained from WWTP at Koprivnica, Croatia, in concrete and mortar mixtures. SWWTS was used as partial replacement of cement (10 and 20%) as produced, and after thermal treatment at temperatures of 800, 900 and 1000 °C. It was shown that the SWWTS particles ranged between 5 and 500 µm, with most particle sizes between 20 and 63 µm. The main crystalline phases were calcite (CaCO_3_) and portlandite (Ca(OH)_2_), while secondary crystalline phases were anhydrite (CaSO_4_), dolomite (CaMg(CO_3_)_2_) and illite. Results showed that replacement of cement with the SWWTS led to the reduction in compressive and flexural strength and increase in permeability and water penetration depth when compared to the reference concrete. 

Within the research presented in this paper, SWWTS produced in Belgrade, Serbia, was applied as a partial replacement of cement in lightweight pervious concrete mixtures. Prior research suggested that WWTS addition leads to an increase in water demand influencing the consistency of the fresh mix [30,31], whereas the SWWTS addition decreases mechanical properties of ordinary concrete [27]. For every 5% increase on average in WWTS ash content in mortars, the fluidity decreases by 4.07% [32]. It was also found that porosity of mortar increases by 3.73% on average, for every 20% increase of WWTS ash content in cement mortars [32]. Therefore, considering previous findings, LWPCP was selected as a product where the SWWTS was incorporated in the cement matrix.

## 3. Materials and Methods

### 3.1. Materials

Four series of samples were produced, one reference mixture, and three mixtures where cement was replaced by 10, 20 and 30% by mass. In order to understand the effects of its addition to the cement matrix, the primary step of this study was to conduct physical and chemical analysis on the SWWTS as produced, and then thoroughly investigate the obtained LWPCP samples at the age of 28 days.

In the production of all tested concrete mixtures, cement CEM I 52.5 R was used. Expanded clay aggregate with a grain size between 1 and 4 mm in diameter was employed as aggregate in all mixtures. Expanded clay aggregates have a closed pore structure, low bulk density and thermal conductivity. According to the declaration of performance, loose bulk density of this aggregate was 450 ± 65 kg/m^3^ with water absorption after 24 h of 11 ± 4%. Tap water was used in all mixtures, together with air-entraining and accelerating admixtures.

SWWTS was used as a partial replacement of cement in the amounts of 10, 20 and 30% by mass. This material is a light-gray (grayish) powder with hydrophobic properties (detailed characterization is presented in Chapter 4).

The amount of water remained the same in all of the prepared mixtures, so that the water/(cement + SWWTS) ratio remained constant, but the water/cement ratio was increased, from 0.300 in the reference mixture, to 0.428 in the mixture with 30% cement replacement.

**Figure 1:**
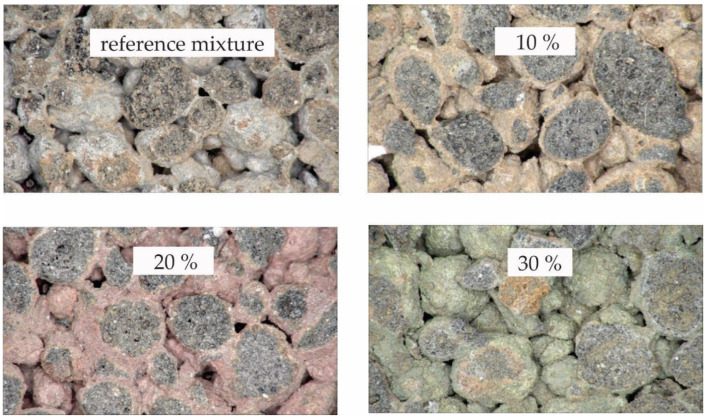
Images of prepared pervious concrete samples (30× magnification).

Within the presented study, four series of lightweight pervious concrete pavers were prepared. The images of representative samples are presented in Figure 1, with magnification of 30×. The amounts (in kg/m^3^) of cement, SWWTS, expanded clay aggregate and water are presented in Table 1. In order to distinguish the samples by color, different pigments were added to each mixture, as described in Table 1. 

### 3.2. Methods

**Figure 2:**
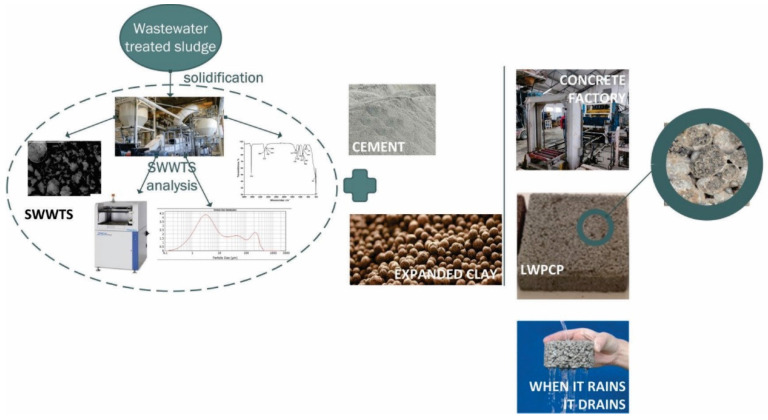
The schematic representation of the performed research.

The presented research was divided into two phases, as shown in Figure 2. Firstly, detailed characterization of SWWTS was performed, in order to evaluate its properties related to application as SCM. Secondly, LWPCP samples were prepared, as explained above, and their physical and mechanical properties were investigated. 

Particle size and particle size distribution were measured by the laser light scattering method using Mastersizer 2000 (Malvern Instruments, Malvern, UK). Furthermore, particle size distribution of powdered samples was measured using Mastersizer Scirocco 2000 analyzer (Malvern Instruments, UK). The results obtained are presented through three dependent parameters: surface weighted mean diameter (SD) (µm) or volume weighted mean diameter (VD) (µm), specific surface area (SSA) (m^2^/g) and span values.

Chemical composition of SWWTS was measured through the energy dispersive X-ray fluorescence (XRF) characterization; using XRF spectrometer produced by Xepos, Spectro with a binary cobalt/palladium alloy thick-target anode X-ray tube (50 W/60 kV) and combined polarized/direct excitation. Air was used for the cooling system. The same equipment was used for determination of heavy metals content in the specimen. Before testing, samples were dried until constant mass at 105 °C. After drying, they were prepared as pressed pellets (40 mm in diameter and 3 mm in height) by mixing coal and tableting aid wax. 

Crystalline phases were identified by X-ray powder diffraction (XRD) using Rigaku MiniFlex 600 diffractometer, Rigaku Corporation, Tokyo, Japan (CuKα radiation, *λ* = 0.15406 nm; 2*θ* = 10–70°; scan rate = 0.02 s^−1^).

Through coupled SEM-EDS technology, morphology of samples and chemical composition of selected areas was determined. Testing was performed on Jeol JSM5800 SEM with a SiLi X-Ray detector (Oxford Link Isis series 300, Oxford, UK), using magnifications of 200, 1000, 3000 and 9000.

The Fourier transform infrared spectroscopy (FTIR) was used for qualitative analysis of functional groups of SWWTS samples. For this purpose, ATR FTIR-Fourier transform infrared spectroscopy (FTIR) spectra of the SWWTS sample were recorded in the absorbance mode using a Nicolet™ iS™10 FT-IR Spectrometer (Thermo Fisher SCIENTIFIC, Bremen, Germany) with Smart iTR™ Attenuated Total Reflectance (ATR) sampling accessories, within the range of 400–4000 cm^−1^, at a resolution of 4 cm^−1^ and in 20 scan modes.

Determination of Ca(OH)_2_ and CaCO_3_ in the analyzed sample was conducted by titration of a known volume of liquid sample (after dissolution in distilled water in an ultrasound bath). The titration was performed with a standard solution of H_2_SO_4_ (0.05 mol/dm^3^) using phenolphthalein and methyl-orange indicators, respectively.

In order to obtain representative samples, the production of LWPCP was organized at the concrete prefabrication plant, using the existing production line, with the necessary equipment for vibration and cutting of samples. Nominal dimensions of the pavers were 200 × 200 × 60 mm. They were cured in laboratory conditions (temperature 20 ± 2 °C, relative humidity 50 ± 10%) up to the age of 28 days, when the tests were performed.

Bulk density was measured as average value of three specimens for each type of concrete. Volume of the specimens was determined through measurements of their dimensions using a digital caliper with 0.01 mm accuracy and scale of 0.1 g accuracy. 

Water absorption was determined through measurements of samples dried to constant mass, and then gradually immersed in water until reaching the constant mass. It was measured as an average value recorded on two tested samples.

Mercury intrusion porosimetry (AutoPore IV 9500, Norcross, GA, USA, Micromeritics) was used to analyze the pore size distribution and porosity. Maximal intrusion pressure used was 228 MPa.

Saturated hydraulic conductivity and thermal conductivity were determined only for the reference mixture. A constant head permeability test is the standard method for determination of the PCPs hydraulic conductivity [33,34]. Thermal conductivity measurements were performed according to EN 12667, using guarded hot plate apparatus, with a measurement error lower than 1%.

Flexural strength and ultrasonic pulse velocity measurements were performed on the prismatic samples that were cut out of the pavers. The dimensions of the prisms were 200 × 60 × 60 mm. Flexural strength was determined through a three-point-bending test, using a span of 150 mm. Breaking force was measured to 0.1 kN accuracy. For the ultrasonic test, samples were additionally flattened from the sides with a thin layer of cement mortar. Ultrasonic testing equipment, with probes frequency of 24 kHz, was used. Both properties were calculated as average values of three tests. 

Compressive strength was measured on the samples cut out from the original pavers. Their nominal dimensions were 60 × 60 × 60 mm. During the test, the force was transmitted on the samples through steel plates 40 mm wide. Compressive strength was calculated as a ratio of the force measured at breakage and the area of the load transfer as an average value of six measurements. Force was measured with an accuracy of 0.1 kN.

Pull-off tester, with a range between 0 and 16 kN, and accuracy of 0.01 kN, was used for pull-off strength measurements, according to EN 1542. Due to the possible damaging effect, steel plates were only glued to the testing surface, with no additional cutting. Adhesion strength was determined as a ratio between braking force and the fractured surface. It was calculated as an average value of three measurements.

## 4. Results

### 4.1. Chemical Composition of Wastewater Treatment Sludge (WWTS)

Wastewater treatment sludge was analyzed for heavy metal content, before and after the solidification treatment. 

### 4.2. SWWTS Characterization

#### 4.2.1. Particle Size Distribution

**Figure 3:**
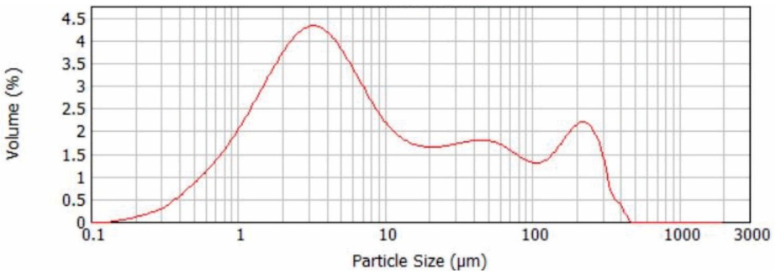
Particle size distribution of the SWWTS sample.

Particle size distribution of the SWWTS sample is presented in Figure 3. From the diagram it can be observed that the distribution curve is polymodal, suggesting multiple most commonly occurring sizes of the particles. The most intense peak implied the presence of particle sizes ranging from 3 to 5 μm; furthermore, particle sizes from 200 to 250 μm were also detected. An additional shoulder peak revealed the presence of particle sizes from 40 to 50 μm. 

In Table 2, dependent parameters obtained from the analysis of particle size distribution are presented. From the presented parameters, it can be concluded that the percentage of particles smaller than 1.103 µm was 10% (D_10_), the percentage of particles smaller than 5.814 µm was 50% (midpoint D_50_) and that the percentage of particles smaller than 158.74 µm was 90% (D_90_). Midpoint D_50_ is the size that splits the particle size distribution with half above and half below this diameter. The D-value is one of the easier statistics to understand as well as the most meaningful for particle size distributions [35].

The span parameter is an additional parameter that shows the width of the size distribution and the volume-based size distribution is defined as: Span = (D_90_–D_10_)/D_50_. Span parameter indicates how far the 10 and 90 percent points are apart, normalized with the midpoint [35,36]. 

**Figure 4:**
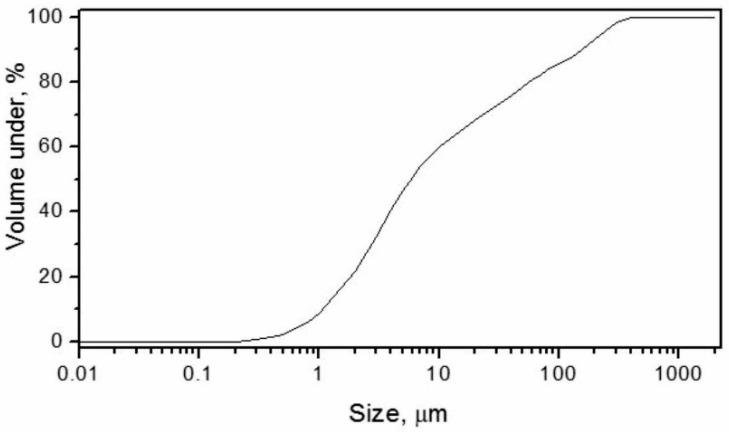
The cumulative particle size distribution curve for the SWWTS sample.

These finding are in correlation with the median parameter obtained from the cumulative distribution curve (Figure 4), where the percentage of the most commonly occurring particle size is presented. 

One of the main properties of any material used as SCM is its fineness, no matter if it is used as a pozzolanic addition or filler. Almost 60% of the tested sample showed particle diameters lower than 10 µm, which encourages its use as an SCM. The particles, with diameters higher than 100 µm, should be considered for additional milling in the future. In order to avoid additional energy consumption through milling, the SWWTS was used in “as received” state. 

#### 4.2.2. X-Ray Fluorescence (XRF)

Heavy metals content was determined in order to confirm that the tested material presents a nonhazardous waste. The results are shown in Table 3. The amounts of the elements are very small, between 2 and 20 times smaller than before solidification treatment.

Results of the XRF measurements are shown in Table 4. 

As expected for the solidified WWTS, CaO is the main constituent of the tested sample. Total amounts of SiO_2_, Al_2_O_3_ and Fe_2_O_3_ are extremely low, less than 1%, which confirms that SWWTS cannot be treated as a pozzolanic SCM, but rather only as a filler. 

#### 4.2.3. XRD Analysis 

**Figure 5:**
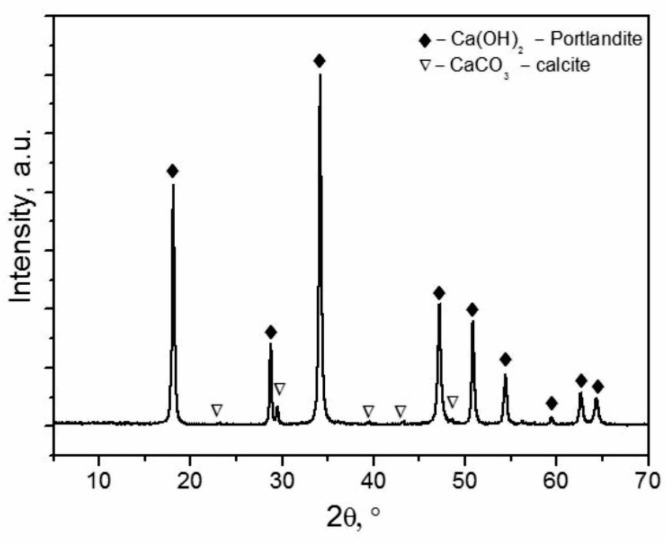
X-ray diffraction patterns of the SWWTS sample.

XRD analysis showed that the SWWTS sample (Figure 5) consists dominantly of calcium hydroxide crystalline phase, exhibiting a typical Ca(OH)_2_ portlandite pattern with sharp, pronounced high intensity peaks that correspond to JCPDS 01-073-5492 [37,38]. An additional minor crystalline phase with wide low intensity peaks was also detected that can be attributed to the CaCO_3_ calcite phase (JCPDS 24-0027) [39]. 

#### 4.2.4. SEM

**Figure 6:**
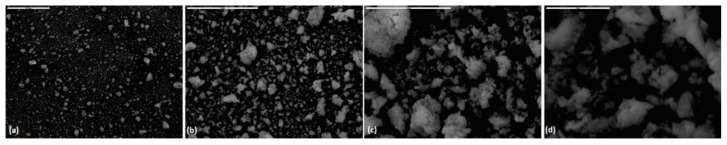
SEM images of the powder Ca(OH)_2_–CaCO_3_ complex aggregates with different magnification: (**a**) 200; (**b**) 1000; (**c**) 3000; and (**d**) 9000.

From the SEM images presented in Figure 6, complex aggregates of Ca(OH)_2_ and CaCO_3_ particles can be observed. At higher magnifications (Figure 6c,d), individual agglomerates can be observed consisting of numerous, irregular smaller particles [40]. These SEM images revealed higher presence of the granular stone shaped that can be attributed to the Ca(OH)_2_ [41].

**Figure 7:**
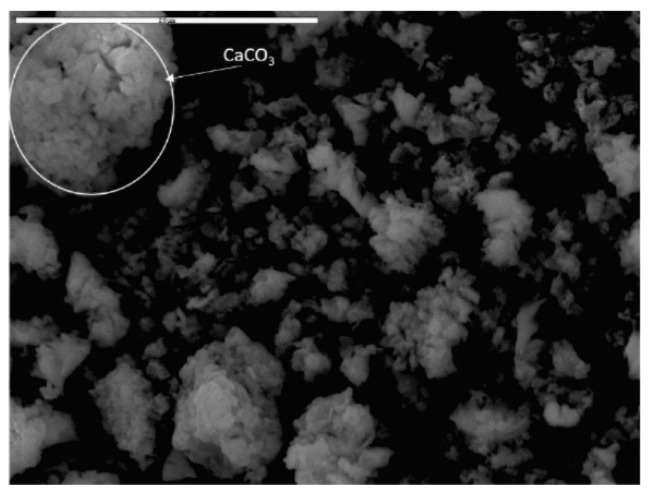
SEM images of the powder Ca(OH)_2_–CaCO_3_ complex aggregates with CaCO_3_ particle detection.

Additionally, multiple layered porous hierarchical flake-like structures with relatively large grain sizes were also observed, which could be related to the lower presence of CaCO_3_ particles [41] (see Figure 7). These results are in accordance with the XRD analysis as well as with the chemical analysis where substantially higher Ca(OH)_2_ phase content and amount was detected. 

SEM and EDS analyses were used to investigate surface morphology and physico-chemical structure of the solid particle surface in SWWTS sample. It can also present the information about the nature of the particles. From the results, it was confirmed that the SWWTS consists of inorganic matter, even though it was obtained by the treatment (solidification) containing mostly organic sludge. 

The presence of O, Mg, Si, K and Ca, with prevailing Ca and O content, were confirmed by EDS spectrum as presented in Table 5. These results are also in accordance with the previous presented chemical and structural characterization of the SWWTS sample. 

#### 4.2.5. Fourier Transform Infrared Spectroscopy–FTIR

**Figure 8:**
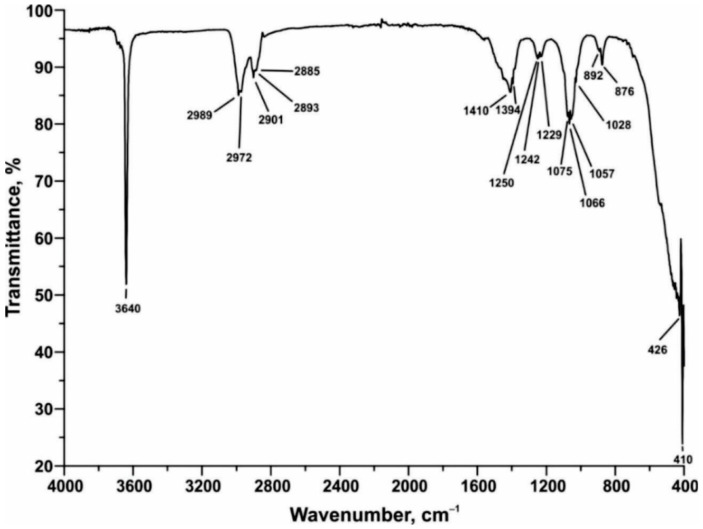
Obtained FTIR spectra of SWWTS sample.

The FTIR spectra of SWWTS sample is shown in Figure 8.

The appearance of absorption bands indicates the presence of the following functional groups: detected bands at 3640 and 1394 cm^−1^ correspond to O-H hydroxyl groups from Ca(OH)_2_, while 2989–2840 cm^−1^ correspond to C-O (organic origin). The absorption bands at 1410, 426 and 410 cm^−1^ propose the presence of CaO in the sample, whereas detected bands at 1066, 892 and 876 cm^−1^ could be associated with C-O from CaCO_3_. Again, the prevalence of calcium-hydroxide and calcium-carbonate was determined from the spectrum. Furthermore, from the FTIR analysis it can be concluded that after the treatment, the nature of the SWWTS material is dominantly inorganic, with only one peak (between 2989–2840 cm^−1^) indicating a slight presence of matter with organic origin.

#### 4.2.6. Chemical Titration

As it was shown through the XRF, XRD, SEM and FTIR analysis, the tested sample showed high calcium content. In order to determine which calcium compound prevails, chemical titration was used. It was shown that the SWWTS sample consisted mainly of the Ca(OH)_2_, with concentration of 89.5%. CaCO_3_ content was 6.1% in the analyzed sample.

### 4.3. Physical and Mechanical Properties of LWPCPs 

#### 4.3.1. Bulk Density

**Figure 9:**
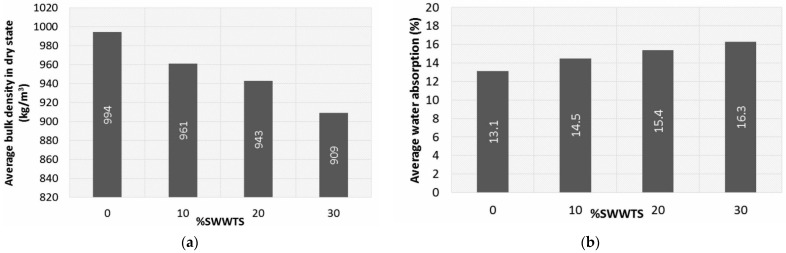
(**a**) Bulk density of tested mixtures; (**b**) water absorption of tested mixtures.

As is presented in Figure 9a, the addition of SWWTS as a partial replacement of cement leads to the reduction in the bulk density. Nevertheless, the differences are lower than 10% (3% for the mixture with 10% cement replacement and 8.5% for the mixture with 30% cement replacement). 

#### 4.3.2. Water Absorption 

Water absorption results are presented in Figure 9b. The increase in the percent of cement replacement with SWWTS led to the increase in water absorption, by approximately 1% for 10% cement replacement.

#### 4.3.3. Ultrasonic Pulse Velocity

**Figure 10:**
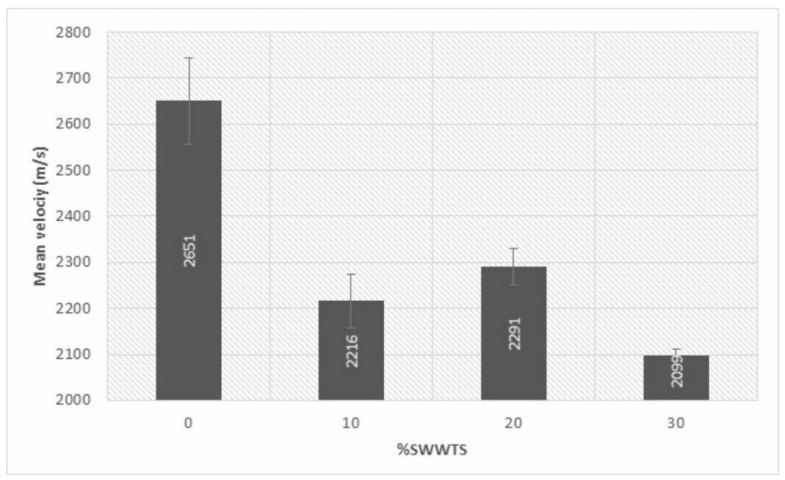
Ultrasonic pulse velocity of tested mixtures.

Ultrasonic pulse velocity was used as a nondestructive method to provide an additional parameter to the density, porosity and microstructural measurements of the tested materials. As is shown in Figure 10, the partial replacement of cement led to a decrease in the ultrasonic pulse velocity. 

By carefully examining the data, it was found that both bulk density and water absorption changed linearly with the increase in SWWTS. In the case of ultrasonic pulse velocity, a higher value was unexpectedly reached for the mixture with 20% cement replacement when compared to the mixture with 10% replacement. When standard deviations were taken into account, the findings seem to suggest that the detected difference could be the consequence of the heterogeneity of samples. 

#### 4.3.4. Mercury Intrusion Porosimetry (MIP)

**Figure 11:**
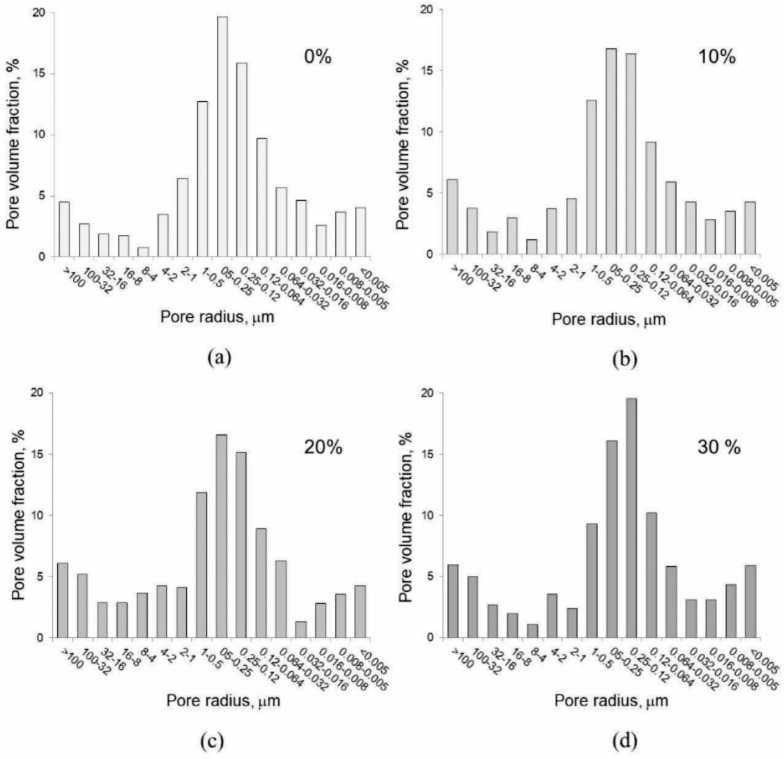
Pore size distribution of studied LWPCP samples measured by Hg porosimetry: (**a**) reference sample with 0% cement replacement, (**b**) sample with 10% cement replacement, (**c**) sample with 20% cement replacement and (**d**) sample with 30% cement replacement.

The results of the mercury porosimetry analysis are given in Table 6 and in Figure 11. All studied samples had similar porosity and pore size distribution. The porosity of all samples is in the range from 34.85 to 37.65%, whereas the cumulative pore volume ranges from 0.2451 to 0.2761 cm^3^/g. The pore size distribution of all samples is multimodal and wide with the predominant fraction of pores in diameter a range from 0.012 to 1 µm (Figure 11). 

Furthermore, all samples also have a small fraction of larger macropores with a diameter larger than 12 µm, and also an indication of a small fraction of smaller pores with the diameter smaller than 0.008 µm. A slight difference in the pore size distribution among the samples can be observed in the broadening of the predominant pore size fraction. Pores with diameter smaller than 1 µm can be attributed to the pores in the hardened cement or cement-SWWTS matrix. From these presented results, it is clear that large macropores that characterize LWPCP cannot be measured by MIP due to small dimensions of the sample. 

#### 4.3.5. Saturated Hydraulic Conductivity and Thermal Conductivity

According to the preliminary results of the ongoing study, the saturated hydraulic conductivity of investigated LWPCPs varies between 1.1 × 10^−2^ and 1.4 × 10^−2^ m/s, which is in compliance with the recommended minimal value, 6.5 × 10^−4^ m/s, for PCPs [9]. The average value of thermal conductivity for the reference mixture was equal to 0.299 W/mK. It is usually considered that the thermal insulation materials with structural properties show a thermal conductivity value ≤0.3 W/mK. Since all of the tested mixtures had similar values of cement paste porosity, and since the addition of SWWTS should not influence macro porosity, saturated hydraulic conductivity and thermal conductivity should have similar values for the mixtures containing SWWTS, as well. These tests will be performed in the continuation of the research.

#### 4.3.6. Mechanical Properties 

**Figure 12:**
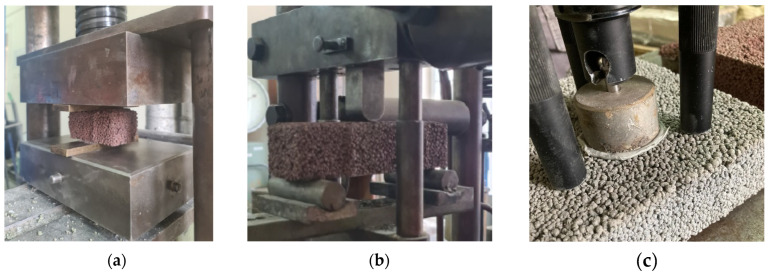
Dispositions of the mechanical properties testing: (**a**) compressive strength, (**b**) flexural strength and (**c**) pull-off strength.

**Figure 13:**
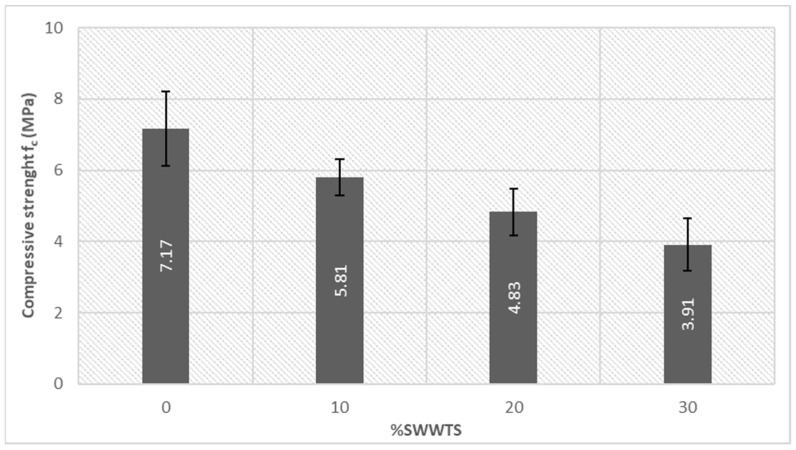
Compressive strength of tested mixtures.

**Figure 14:**
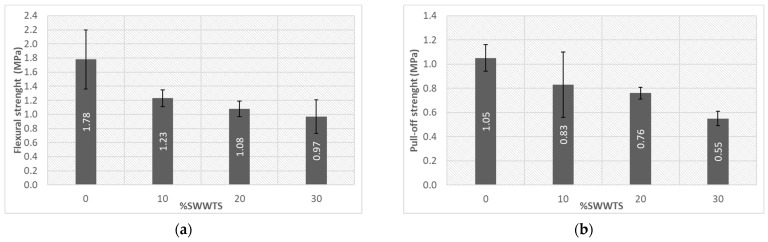
(**a**) Flexural strength; (**b**) Pull-off strength of tested mixtures.

Dispositions of the mechanical properties testing are shown in Figure 12, while the average values of the strength with calculated standard deviations are presented in Figure 13 and Figure 14. 

As shown in Figure 13, partial replacement of cement initiated the decrease in compressive strength values. Although the presented standard deviations of the results are significant, due to the inhomogeneity and porosity of the samples, they do not affect the observed trend. Partial replacement of 30% cement with SWWTS induced a drop in strength of 45.5%, when compared to the reference mixture.

Flexural strength (Figure 14a) of samples revealed a very similar trend: the reference mixture had the strength between the reference mixture and the mixture with 30% cement replacement showed the same difference (drop of strength 45.5%). Similarly, a drop of the pull-off strength (as shown in Figure 14b) was 47.6% for the same mixture. These changes in strength could be explained by the inert nature of the added SWWTS. Compressive strength of the LWPCP should be higher than 5 MPa (for light traffic) according to [9], which was accomplished for the reference mix and mixture with 10% cement replacement. Flexural strength and pull-off strength are not defined in the standard, but their results are valuable in determining the influence of the applied SCM on the mechanical properties of this kind of product. 

## 5. Discussion

Through XRF analysis, as well as SEM-EDS analysis, it was determined that the content of SiO_2_, Al_2_O_3_ and Fe_2_O_3_ was very low in the tested SWWTS sample. Based on these results, and comparing the heavy metal content in samples before and after the solidification treatment, it can be confirmed that during this oxidative process, sludge was transformed not only into nonhazardous, but also nonpozzolanic material. 

XRD analysis revealed that the SWWTS sample consisted dominantly of calcium hydroxide crystalline phase with additional minor crystalline phase that can be attributed to the CaCO_3_ calcite phase. These findings were fully consistent with the results obtained from SEM and FTIR analysis concluding that SWWTS consists mainly of inorganic compounds. Quantity of Ca(OH)_2_ and CaCO_3_ was determined through chemical titration, and it was shown that 89.5% of the sample was Ca(OH)_2_. 

Based on the overall assessment of the results, SWWTS could be used in cementitious composites as a filler or as fine aggregate. Considering that the percentage of particles smaller than 1.103 µm was 10% (D10) and the percentage of particles smaller than 5.814 µm was 50% (midpoint D50), the first option was chosen. Nevertheless, it should be stated that the percentage of particles smaller than 158.74 µm was 90% (D90), indicating a portion of coarser grains that could be additionally milled before application being an obvious shortcoming. However, it was important to investigate the application of this material in order to determine future research routes. Results obtained through particle size distribution analysis showed that the tested sample of SWWTS had finer gradation than that produced using the same technology but in another facility [27]. Further research should be focused on testing samples from different batches in order to determine the possible variations in the particle size distribution, which could affect the application of this material.

The presented research showed that the partial replacement of cement with SWWTS led to the decrease in all mechanical strengths. This reduction was expected, since the SWWTS can only replace the filler role of cement particles, and thus cannot be treated as a binder. In this light, through preservation of the same amount of water, the water/cement ratio increased in mixtures with SWWTS. However, the mixture containing 10% cement replacement reached a compressive strength higher than 5 MPa which means LWPCP complies with EU standards for light traffic (as defined by [9]). The mixture containing 20% cement replacement was also very close to achieving this goal. All of the tested mixtures showed a value higher than 3.5 MPa, which was defined as the lowest strength in the range of pervious concrete properties. According to [33], where SWWTS was used in production of ordinary concrete, the differences in strength of the reference mixture and mixtures containing 10 and 20% of SWWTS, as cement replacement, were higher. The difference in the compressive strength was 40 and 50.9%, respectively, for these two types of mixtures (with untreated SWWTS). In future trials, it could be considered to reduce the amount of water, preserving the same water/cement ratio, in mixtures containing SWWTS in order to try to reduce the decline in strength of these mixtures. Although this type of material is a very dry mix in the fresh state, it has to be investigated if the reduction of water would lead to the complications in the preparation of the samples.

Although it is usually not demanded to test the flexural strength before the application of pervious concrete, the data from the literature suggest that it ranges between 1 and 3.8 MPa [6]. All mixtures except for the one with the highest level of cement replacement fulfilled this requirement. Taking into account the standard deviations of the results, it can be concluded that the introduction of SWWTS in the system led to the decrease in the flexural strength of 30%, but that the differences between the strength of the mixtures with 10% and 20% cement replacement, are very low. Pull-off strength that was tested in order to complement the results of the mechanical tests, also showed a decrease with higher SWWTS content. 

Addition of SWWTS led to a slight decrease in bulk density of the mixtures and increase in water absorption. This can be explained by the reduction in the hydration products that would fill in the micropores of the matrix, since SWWTS showed no pozzolanic reactivity. Still, the porosity measured through MIP showed similar values for all tested mixtures, ranging between 34.6 and 37.6%. Pore sizes that prevail in the tested binder matrices are in accordance with the results measured on ordinary pervious concrete (the largest fraction of pores had a diameter between 0.02 and 0.2 μm) [42]. 

The saturated hydraulic conductivity showed values within expected limits. More testing of the mixtures containing SWWTS will be performed in the future, but in light of the results of the pore size distribution, it is expected that they will reach similar values. Low thermal conductivity recommends LWPCP as potential candidates for rooftop elements. 

## 6. Conclusions

The main objective of the presented research was to determine the possibility of using SWWTS as a partial replacement of cement in LWPCP. Therefore, detailed chemical analysis on the SWWTS was conducted in order to determine its composition and possibilities of application. 

It was shown that the fineness of the tested SWWTS was suitable for its application as SCM, but its chemical composition indicated that it cannot be treated as a pozzolanic addition. All the analyses showed the inorganic nature of this waste product, and confirmed that it is nonhazardous for the environment. 

The addition of the SWWTS to LWPCP mixtures, led to a linear decrease in all mechanical parameters, bulk density and ultrasonic pulse velocity and an increase in water absorption. The mixture with 30% of the cement replaced with SWWTS showed a reduction in strength of 45% when compared to the reference mixture. However, the mixture containing 10% of SWWTS showed a compressive strength higher than 5 MPa, which is in line with EU standards for light traffic PCPs.

Since the abrasion resistance and durability aspects are very important for the application of these pavements, there is ample scope for further work regarding these tests. Clearly, the next step should be to explore and test porous concrete mixes, based on normal aggregate with the addition of SWWTS as cement or fine aggregate replacement. Another possibility for application of SWWTS in cement composites is its chemical activation that would enable higher mass ratios in concrete mixtures. 

## Figures and Tables

**Table 1:** Composition of the tested mixtures for lightweight pervious concrete (kg/m^3^).

Material	Reference Mixture	10%	20%	30%
Cement CEM I 52.5R	300	270	240	210
SWWTS	0	30	60	90
Expanded clay–1/4 mm	720	720	720	720
Water	90	90	90	90
Pigment	Gray	Brown	Red	Green
Air-entraining admixture, Accelerating admixture				

**Table 2:** Parameters obtained from the analysis of particle size distribution.

Parameter	SWWTS
Specific surface area (m^2^/g)	2.190
Surface Weighed mean D(3,2)	2.740
Volume Weighed mean D(4,3)	41.282
Span	27.111
D_10_ (µm)	1.103
D_50_ (µm)	5.814
D_90_ (µm)	158.74

**Table 3:** Heavy metals content before (WWTS) and after the solidification treatment (SWWTS).

Element	WWTS (mg/kg)	SWWTS (mg/kg)
Cl	No data	387.10
Ni	No data	16.18
Cu	44.5	6.21
Zn	120	13.10
As	20.2	0.10
Sr	No data	121.90
Mo	No data	1.91
Cd	<0.6	0.30
Ba	56.6	27.80
Pb	13.1	1.31

**Table 4:** The quantitative results of XRF analysis.

	Content (%)
L.O.I. at 1000 °C	26.93
SiO_2_	0.14
Al_2_O_3_	0.14
Fe_2_O_3_	0.03
CaO	71.70
MgO	0.51
Na_2_O	0.01
K_2_O	0.07
SO_3_	0.27
Others	0.20

**Table 5:** The quantitative results of EDS analysis.

Content (%)	S1	S2	S3	Average
O	50.1	53.1	52.3	51.8
Mg	0.3	0.3	0.3	0.3
Si	0.1	0.2	0.2	0.2
K	0.1	0.1	0.2	0.1
Ca	45.7	43.2	43.6	44.2
Others	3.7	3.1	3.4	3.4

**Table 6:** The porosity and cumulative pore volume of samples obtained by mercury porosimetry.

Sample, % SWWTS	Porosity (%)	Cumulative Pore Volume (cm^3^/g)
0	37.65	0.2761
10	34.85	0.2451
20	34.58	0.2476
30	36.78	0.2759

## Data Availability

Not applicable.
